# Genome-wide association study of salicylic acid provides genetic insights for tea plant selective breeding

**DOI:** 10.1093/hr/uhae362

**Published:** 2025-01-02

**Authors:** Xiuling Deng, Dejun Huang, Yihan Wang, Hongwei An, Dingchen Bai, Xiaojing Wang, Suzhen Niu, Xiaoming Song

**Affiliations:** College of Tea Science/Institute of Agro-Bioengineering, Guizhou University, Jiaxiu South Road, Huaxi District, Guiyang 550025, China; College of Tea Science/Institute of Agro-Bioengineering, Guizhou University, Jiaxiu South Road, Huaxi District, Guiyang 550025, China; Department of Tea Studies (Guizhou Duyun Maojian Tea College), Guizhou Vocational College of Economics and Business, Green Lake Industrial Park, Duyun 558000, China; College of Tea Science/Institute of Agro-Bioengineering, Guizhou University, Jiaxiu South Road, Huaxi District, Guiyang 550025, China; College of Tea Science/Institute of Agro-Bioengineering, Guizhou University, Jiaxiu South Road, Huaxi District, Guiyang 550025, China; College of Tea Science/Institute of Agro-Bioengineering, Guizhou University, Jiaxiu South Road, Huaxi District, Guiyang 550025, China; The Key Laboratory of Plant Resources Conservation and Germplasm Innovation in the Mountainous Region (Ministry of Education), Xueshi Road, Huaxi District, Guiyang 550025, China; College of Tea Science/Institute of Agro-Bioengineering, Guizhou University, Jiaxiu South Road, Huaxi District, Guiyang 550025, China; The Key Laboratory of Plant Resources Conservation and Germplasm Innovation in the Mountainous Region (Ministry of Education), Xueshi Road, Huaxi District, Guiyang 550025, China; School of Life Sciences/School of Basic Medical Sciences, North China University of Science and Technology, Tangshan 063210, China

## Abstract

Salicylic acid (SA) is a phenolic phytohormone widely believed to regulate plant growth and stress response. Despite its significance, the genetic basis of SA-mediated resistance to biotic stressors in tea plants is little understood. Our study investigated the genetic diversity, population structure, and linkage disequilibrium (LD) patterns of 299 tea accessions using 79 560 high-quality single nucleotide polymorphisms (SNPs) obtained from genotyping-by-sequencing (GBS) data. Our genome-wide association study identified *CSS0033791.1*, an essential gene encoding 9-cis-epoxycarotenoid dioxygenase (CsNCED1), which catalyzes a vital step in abscisic acid (ABA) biosynthesis. Exogenous ABA treatment and transgenic overexpression of the *CsNCED1* gene lowered SA content in the respective tea plants by inhibiting the expression of the *ICS* gene. Further analysis revealed that ABA could reduce the expression levels of the SA receptor gene (*NPR1*) and NPR1 target genes (*PR1* and *WRKY18*), increasing the plant’s susceptibility to biotic stressors. Furthermore, the feeding behavior of *Spodoptera litura* revealed that the insect bite area on transgenic leaves was substantially more extensive than that in wild type (WT), implying that the *CsNCED1* gene had a negative regulatory role in SA-mediated immune response. This study thus provides the foundation for future insect resistance breeding, sustainable tea plant resource usage, and molecular marker-assisted (MAS) tea plant breeding.

## Introduction

The tea plant originated in southwest China and is used to make one of the world’s top three nonalcoholic beverages. It holds significant economic, social, and cultural value [1–3]. However, tea plants are susceptible to pests and diseases, resulting in substantial crop losses [4]. The phytohormones salicylic acid (SA), abscisic acid (ABA), jasmonic acid (JA), and ethylene have been reported to regulate plant growth and respond to various biotic stressors via complex signaling pathways, particularly SA [5–8]. However, the genetic mechanisms underlying SA’s role in the immune response to biotic stresses in tea plants are poorly understood.

As a defense hormone, SA has been linked to increased immunity against biotrophic and semibiotrophic pathogens [9, 10]. It is essential for basal defense, local immune responses, and the systemic defense response. SA is present in the cytoplasm and interacts with other hormones to control how plants respond to various biotic stressors. Accumulating evidence has indicated that SA interacts with ABA to fine-tune the control of plant–pathogen interactions. ABA acts as a vital positive regulator downstream of SA, creating natural pathways for pathogen entry during preinvasive lesions [11–14]. However, ABA can impede SA biosynthesis and signaling transduction during pathogen infections. SA biosynthesis occurs via two pathways: the PAL and the ICS (the primary path). NPR1, the primary receptor of SA signaling, regulates most SA-mediated pathogenesis-related genes that contribute to plant–pathogen interactions [15].

In recent decades, various indicators, including molecular, morphological, cytological, and biochemical markers, have been extensively used to study the genetic diversity of tea resources [16]. Whole-genome resequencing technology can provide more comprehensive variation data by analyzing structural differences between various individual genomes, completing annotation after sequencing individuals, and comparing sequences. However, it is relatively expensive. Genotyping-by-sequencing (GBS), on the other hand, is a cost-effective approach used to detect high-quality single nucleotide polymorphisms (SNPs) and investigate genetic diversity, kinship, and genetic variation across numerous plant species [17–19]. Genome-wide association studies (GWAS) have become an effective method for identifying the genetic variants associated with specific phenotypes [20]. GWAS has been extensively used to predict genes for tomato traits [21], wheat agronomic traits [22], and rice diseases [23]. Recently, GWAS has been used to identify genetic variations and essential genes related to significant agronomic traits within tea plant germplasm [24]. For example, wild tea plants are resistant to various diseases, including anthracnose, white star disease, tea blister blight, and leaf blight [25, 26]. However, the genetic variation contributing to these resistances in tea plants is yet to be documented. So far, GWAS has not been used for SA association mapping in tea accessions.

The present study identified and analyzed high-quality SNPs of 299 tea plant germplasms (collected in Guizhou) using GWAS to investigate the relationships between dynamic fluctuations in SA content and genetic variants. The findings would provide a solid theoretical and material framework for local insect resistance breeding and sustainable utilization of local tea plants in Guizhou, promoting the rapid development of MAS breeding of insect-resistant varieties.

## Result

### Analysis of SA content

**Fig. 1:**
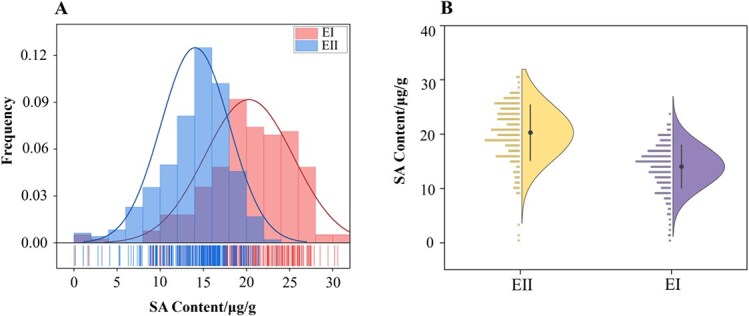
**Frequency distribution of SA content across 299 tea accessions under the two environments.** (A and B) Two-environment frequency distribution map of SA content across 299 tea accessions varieties. Solid circles and vertical lines represent the mean plus or minus standard deviation lines. Note: * denotes *P* < 0.05; DF: degree freedom; SS: Stdev square.

This study assessed the SA content of 299 tea accessions under two different environments (Table 1). Table 1 shows that SA contents varied from 0.17 to 23.59 μg/g in Environment I (EI) and 0.06 to 30.58 μg/g in Environment II (EII). The coefficient of variation in the SA content was 23.13% in EI and 22.36% in EII. In both environments, the frequency distribution of SA content across the accessions followed a Gaussian or near-Gaussian distribution (Fig. 1).

**Table 1:** SA content analysis for 299 tea accessions under the two environments

Environment	Max (μg/g)	Min (μg/g)	Mean ± SD	CV (%)
EI	23.59	0.17	14.06 ± 3.97	23.13
EII	30.58	0.06	20.29 ± 5.13	22.36

**Note**: CV: coefficient of variation; SD: standard deviation.

### Analysis of population structure and LD

**Fig. 2:**
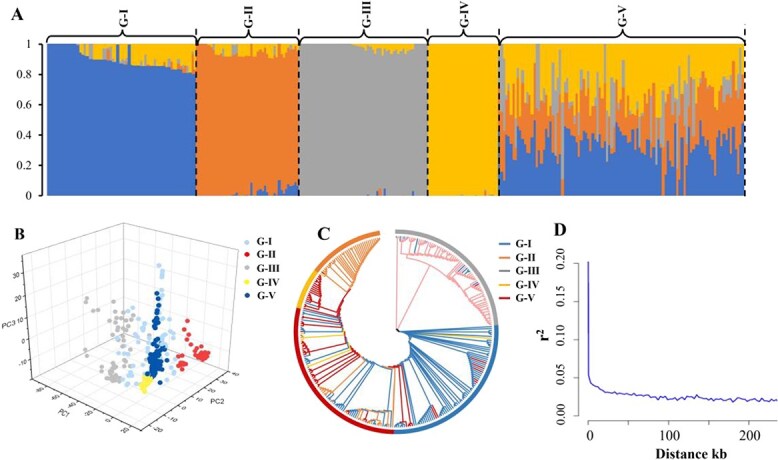
**Population structure analysis of 299 tea accessions using 79 560 high-quality SNPs.** (A) Using Admixture software, 299 tea accessions were divided into five subgroups (K = 4), including G-I–IV, and G-V. (B) PCA of 299 tea accessions. (C) Construction of the NJ phylogenetic tree. (D) LD attenuation diagram.

GBS of 299 tea accessions yielded 29 393 327 SNPs. After applying missing data thresholds, 79 560 high-quality SNPs distributed across the entire genome were obtained, which were then used to perform population structure analysis with Admixture software (Fig. S1, Table ,S3, and  S4). The results indicated that the smallest cross-validation error was detected at K = 4 (Fig. S2). The population structure analysis classified the 299 tea accessions into five subgroups, including four pure subgroups (G I–IV) and one mixed subgroup (G–V), as illustrated in Fig. 2a. PCA validated this division by demonstrating considerable overlap between the subgroups (Fig. 2b and Table S5). An unrooted phylogenetic tree also divided the accessions into five subgroups, which aligned with the results of PCA and population structure analysis (Fig. 2c). Group G-I consisted of 65 accessions representing three species (51 *C. sinensis*, 9 *C. tachangensis*, and 5 *C. remotiserrata*). Group G-II contained 44 accessions, all of which were *C. sinensis*. Group G-III comprised 55 accessions from three species (28 *C. tachangensis*, 25 *C. remotiserrata*, and 2 *C. taliensis*). Group G-IV consisted of 30 accessions comprising two species (29 *C. sinensis* and 1 *C. remotiserrata*). The G-V group contained 105 accessions from four species (79 *C. sinensis*, 15 *C. remotiserrata*, 9 *C. tachangensis*, and 2 *C. taliensis*).

LD analysis, represented by r [2], indicated relatively short r^2^ distances (~15 kb) and rapid decay in LD, with r^2^ values halving at a distance of 15 kb (Fig. 2d).

### Analysis of genetic diversity

Genetic diversity reflects the balance between the emergence and disappearance of genetic variants (alleles). We used Plink v. 1.90 to determine observed heterozygosity (*Ho*), inbreeding coefficient (*Fis*), minor allele frequency (*MAF*), and nucleotide diversity (*Pi*) for each inferred population. Table 2 shows the overall values for the 299 tea accessions: *Ho* = 0.0762, *Fis* = 0.6651, *He* = 0.2168, *MAF* = 0.1378, and *Pi* = 0.0762. Subgroup G-I had a considerably higher *Fis* value than the other subgroups, whereas subgroup G-III had significantly higher *Pi*, *He*, and *MAF* values than the other subgroups. *Pi*, *He*, *MAF*, and *Fis* values were considerably lower in the G-II subgroup compared to the other subgroups. Our results revealed that the five inferred tea subgroups exhibited positive Tajima’s D values, indicating the presence of tea population bottlenecks and/or balanced selection (Table 2).

**Table 2:** The genetic diversity of inferred groups from 299 tea accessions

Group	Number	Tajima’s D	*Pi*	*Ho*	*He*	*MAF*	*Fis*
G-I	65	0.1715	0.1885d	0.0421e	0.1863d	0.1198d	0.7846a
G-II	44	0.1871	0.1730e	0.0801d	0.1708e	0.1140e	0.5320c
G-III	55	0.7242	0.2370a	0.0819c	0.2342a	0.1650a	0.6667b
G-IV	30	0.2270	0.1968c	0.0874a	0.1933c	0.1292c	0.5472c
G-V	105	0.5334	0.2108b	0.0840b	0.2097b	0.1348b	0.6002b
All	299	1.0144	0.2172	0.0762	0.2168	0.1378	0.6651

**Note**: Different lowercase letters indicated significant differences across groups (*P* = 0.05).

### GWAS identifies SNPs and candidate genes associated with SA content

**Fig. 3:**
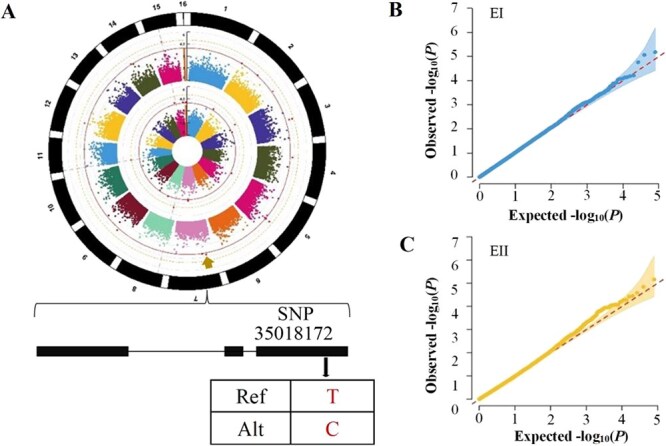
**GWAS for the SA content phenotype and genotype in 299 tea accessions**. (A) Manhattan plots of two environments: the inner circle represents the Manhattan map for EI, the middle circle represents the Manhattan map for EII, and the outer circle represents chromosomes. (B, C) Q-Q plots for the two environments.

**Fig. 4:**
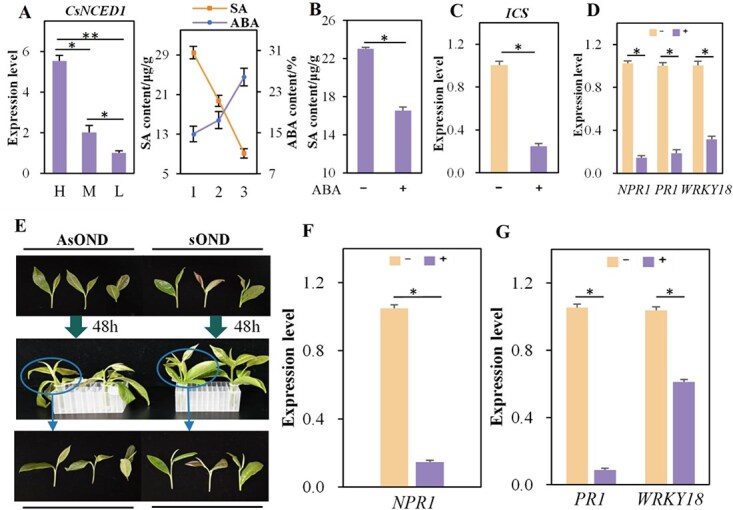
**Antagonistic interactions between ABA and SA responses.** (A) Expression levels of the *CsNCED1* gene (left) and SA and ABA content (right) in three distinct tea plants with high, medium, and low SA levels. ‘H’ indicates high SA content, ‘M’ indicates moderate SA content, and ‘L’ indicates low SA content. (B) Determination of SA content in tea plants treated with exogenous ABA. ‘−’ stands for CK, whereas ‘+’ represents the ABA treatment group. (C) Expression patterns of *ICS* gene in tea plants treated with exogenous ABA. (D) Expression patterns of SA signal transduction genes (*NPR1*, *PR1*, and *WRKY18*) in tea plants treated with exogenous ABA. (E) Phenotypic alterations of young shoots treated with asOND-NPR1. (F) *NPR1* expression levels in young shoots treated with asOND-NPR1. (G) *PR1* and *WRKY18* expression levels in tea plants treated with asOND-NPR1. ‘−’ represents the control group, whereas ‘+’ represents the asOND-NPR1 group.

We conducted GWAS using two separate linear regression models, GLM-P and CMLM-PK, to identify candidate SNPs associated with SA content. We identified 28 significant high-quality SNPs (−log_10_(*P*) ≥ 4.0) [27]. These SNPs accounted for 8.64%–14.24% of phenotypic variance, with estimates ranging from 5.4 to 12.98 for major alleles and − 16.7 to 11.14 for minor alleles (Fig. 3, Table S6). LD analysis resulted in the reannotation of five genes within a 15-kb region surrounding these 28 SNPs using various databases, including NCBI, TPIA, and KEGG (Table S7). The gene *CSS0033791* emerged as a primary candidate for influencing SA content, with an expression pattern contrasting with the SA content dynamics (Fig. 4a, Table S8). This gene contains a leading SNP within a well-defined LD block. It is homologous to *Arabidopsis 9-cis-epoxycarotenoid dioxygenase 1* (*AtNCED1*), which catalyzes the initial step in ABA biosynthesis and influences plant growth, development, and stress responses. However, its specific role in tea plant immune response is still not fully understood.

Further research into the *CsNCED1* gene was conducted by evaluating its expression levels and ABA content in tea plants grown in high, medium, and low SA concentrations (designated as H, M, and L, respectively) (Fig. 4a). The results showed a positive correlation between *CsNCED1* expression levels and ABA content but an inverse correlation with SA content. These findings suggest that *CsNCED1* participates in ABA biosynthesis, which may negatively influence the SA-mediated immune response in tea plants under biotic stress.

### ABA inhibits SA biosynthesis

Several studies have shown that ABA and SA have antagonistic interactions. This study evaluated the effects of exogenous ABA on SA accumulation in tea plants with high SA levels. The results demonstrated that exogenous ABA significantly reduced SA content relative to the control group (Fig. 4b), implying that ABA can inhibit SA accumulation. Pathogen-induced SA is primarily produced by isochorismate synthase (ICS), which is recognized by the NPR1 receptor to regulate plant tolerance to biotic stress. We examined the effects of exogenous ABA on the expression patterns of *ICS* and *NPR1* genes. The findings revealed that in tea plants treated with exogenous ABA, transcript abundances of these genes were significantly lower than in the control group (Fig. 4c, d). In addition, we administered asOND targeting the CsNPR1 gene to the apical buds of tea plants to confirm if *CsPR1* and *CsWRKY18* are specific downstream response genes of the *NPR1* gene. This approach aimed to evaluate the *CsPR1* and *CsWRKY18* gene expression patterns (Fig. 4e, f, g). The results indicated that the expression levels of these targeted genes (*CsPR1* and *CsWRKY18*) in the apical buds treated with asOND were significantly lower than in the control group. These data imply that ABA mitigates SA accumulation by downregulating the expression of the *ICS* gene, which downregulates SA-mediated plant defense signaling.

### Transgenic plants overexpressing *CsNCED1* have enhanced susceptibility to biotic stress

**Fig. 5:**
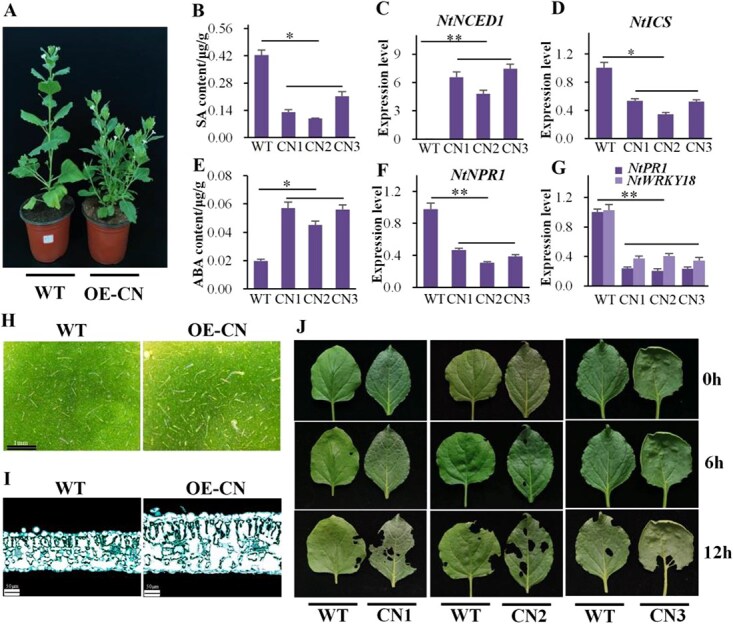
**Overexpression of the *CsNCED1* gene in tobacco.** (A) Phenotypic traits of transgenic tobacco plants overexpressing the *CsNCED1* gene. (B and E) SA and ABA levels in transgenic tobacco plants overexpressing the *CsNCED1* gene. Expression levels of *NtNCED1* (C), *NtICS* (D), *NtNPR1* (F), and *NtPR1*, and *NtWRKY18* (G) genes in three distinct transgenic tobacco lines overexpressing *CsNCED1* gene. (H) A representative figure illustrating the changes in trichome density on transgenic tobacco leaf surfaces. (I) A representative figure illustrating the paraffin sectioning of transgenic tobacco leaves. (J) The feeding behavior of *S. litura* on transgenic tobacco plants versus WT.

We investigated the role of the *CsNCED1* gene in pest resistance by generating transgenic tobacco plants that overexpressed the gene. A total of 11 transgenic lines were successfully developed and verified using PCR analysis. Three lines (CN1, CN2, and CN3) were selected for further study (Fig. 5c). Transgenic tobacco-overexpressing *CsNCED1* exhibited increased growth of lateral branches, denser trichomes on leaf surfaces, higher leaf thickness, and decreased leaf size compared to WT plants (Fig. 5a, h, and i). We also examined the levels of ABA and SA in these transgenic lines. We discovered significantly higher ABA levels and lower SA levels than in WT (Fig. 5b, e), suggesting antagonistic interactions between ABA and SA responses. Transcript abundances of the *ICS*, *NPR1*, *PR1*, and *WRKY18* genes were significantly lower in transgenic tobacco lines than in WT (Fig. 5d, f, and g), consistent with findings from tea plants treated with exogenous ABA. We also conducted insect-feeding experiments on transgenic tobacco plants overexpressing the *CsNCED1* gene. There were no significant differences in insect bite area between WT and transgenic plants 6 h after feeding. However, within 24 h, the bite area on transgenic plants was significantly more extensive than on WT, implying that overexpression of *CsNCED1* may impact insect feeding behavior by reducing SA content (Fig. 5j).

## Discussion

SA is an essential phytohormone-signaling molecule contributing to local- and systemic-acquired resistance against pathogens. Although prior research has underlined the importance of SA in plant immune responses, the specific functions of SA in tea plants’ response to biotic stress are still unclear. In our study, we first systematically evaluated the SA content in 299 individual tea plants, revealing a notable variation in SA content ranging from 0.17 μg/g to 23.59 μg/g in EI and from 0.06 μg/g to 30.58 μg/g in EII. The SA content distribution among the 299 tea accessions followed a normal distribution pattern. We discovered that the SA content varied across tea accessions; nonetheless, the molecular mechanisms governing SA biosynthesis remain unknown.

GBS is an efficient and powerful technique for genotyping breeding populations, enabling the application of GWAS, studying genetic diversity, analyzing genetic linkage, and identifying molecular markers. We used GBS to identify 79 560 high-quality SNPs from 299 tea accessions, unevenly distributed across 15 chromosomes. The results of the population structure analysis showed that the 299 tea accessions were divided into five subgroups: four pure subgroups (G I–IV) and one mixed subgroup (G V). This categorization is consistent with previous studies that classified the tea plant population in Guizhou province into five distinct groups. The accuracy and reliability of our population structure analysis were further supported by the results of the PCA and NJ phylogenetic tree, which consistently grouped tea genotypes into these five clusters.

The GWAS method is widely recognized as a practical approach for investigating the impact of molecular markers on major traits and complex phenotypic features. Recently, GWAS analysis was used on the populations of major crops and horticulture plants to identify the molecular regulatory mechanisms of essential characteristics [17, 28–32]. While GWAS has been frequently employed in tea accessions to reveal molecular regulatory mechanisms of quality and yield, its application to the immune response of tea plants to reveal the putative molecular regulatory mechanism has not been reported. In the GWAS analysis, we used two distinct models (GLM-P and CMLM-PK) to identify SNPs consistently associated with SA content. The study identified 28 high-grade SNPs associated with SA content (−log_10_ (*P*) ≥ 4.0), primarily located on chromosomes 2, 3, 5, 7, 11, 14, and 15, accounting for 10.75% of the variation.

Previous studies have revealed that GWAS utilized the LD decay distance to identify potential candidate gene regions. Xu et al. identified an SNP associated with leaf starch content in *Nicotiana tabacum* using LD decay distance [33]. Rahman et al. screened seven genomic regions associated with quinoa flowering days based on the LD decay distance [34]. Cross-pollinated species, such as tea plants, experienced faster LD decreases than self-pollinated species, which have lower recombination efficiency [35, 36]. We ultimately selected an approximate distance of 15 kb (r^2^ = 0.38) based on LD analysis and identified five candidate genes associated with the SA content. Functional annotation further revealed that one *CSS0033791* gene, encoding 9-*cis*-epoxycarotenoid dioxygenase (NCED), catalyzed a key step in ABA biosynthesis [37, 38]. It is involved in plant responses to various pathogens and exhibits complex antagonistic and synergistic relationships with SA. The results revealed that *CsNCED1* expression and ABA content were significantly lower in high-SA tea plants but substantially higher in low-SA tea plants. These findings indicated an antagonistic relationship between ABA and SA, consistent with previous reports [39–43].

**Fig. 6:**
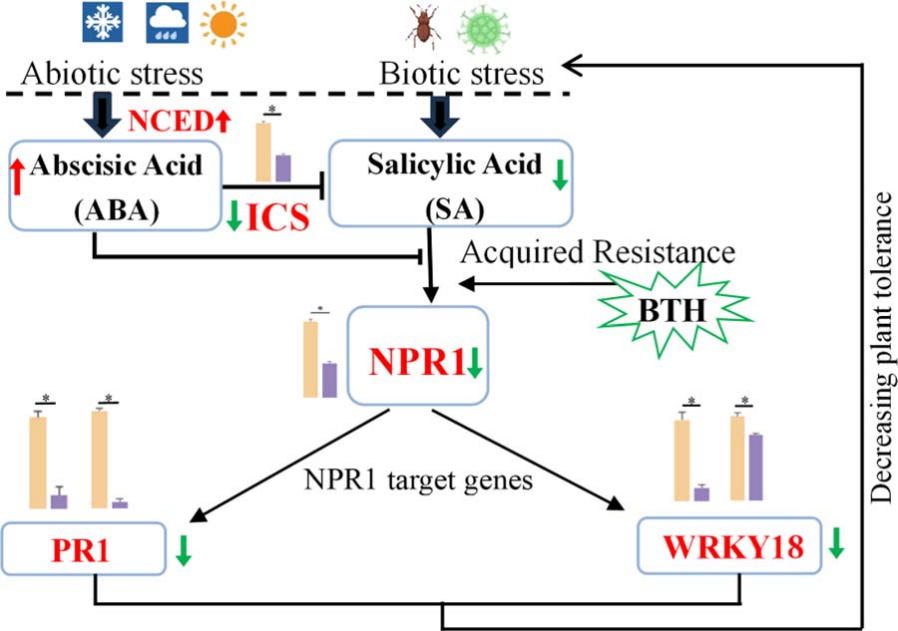
**Proposed model of SA/ABA antagonism.** The bar chart depicts the changes in transcript abundance of the *PR1* and *WRKY18* genes in tea plants treated with ABA and asOND.

Furthermore, the results showed that exogenous ABA significantly suppressed SA accumulation by downregulating the expression of the *ICS* gene, which in turn reduced the expression levels of the NPR1 gene and its downstream target genes (*CsPR1* and *CsWRKY18*). These findings also suggested that ABA was involved in the negative regulation of the SA-mediated immune signal response, which increased susceptibility to biotic stress (Fig. 6). Overexpression of the *CsNCED1* gene in transgenic tobacco lines resulted in significantly higher ABA and lower SA content than WT plants. This conclusion supported the findings of the dynamic variations of ABA and SA in tea plants, as well as the high-SA tea plant treated with exogenous ABA. It confirmed that the *CsNCED1* gene increased ABA biosynthesis while inhibiting SA accumulation in transgenic tobacco plants, consistent with previous reports. Furthermore, transgenic tobacco lines overexpressing the *CsNCED1* gene showed a significant decrease in crucial SA biosynthesis gene (*ICS*), SA receptor gene (*NPR1*), and NPR1 targeted genes (*NtPR1* and *NtWRKY18*). Thus, the *CsNCED1* gene plays a crucial role in the negative regulation of SA-mediated immune signal response. We also conducted an insect-feeding experiment on transgenic tobacco plants overexpressing the *CsNCED1* gene. The results showed that the insect bite area in transgenic tobacco was significantly more extensive than in WT, indicating that *CsNCED1* overexpression may affect insect feeding ability by reducing SA content.

## Conclusion

The present study explored the dynamic distribution of SA content in 299 tea accessions and identified SNPs across the *C. sinensis* genome using the GBS approach. Seventy-nine thousand five hundred sixty high-grade SNPs were identified and used to study population structure, phylogenetic analysis, PCA, and LD in the 299 tea accessions. Additionally, GWAS analysis was used to identify candidate SNPs and genes associated with SA within the linkage group. The results revealed the presence of 28 SNPs and 9 genes linked with SA content, which were distributed across 11 chromosomes. Functional annotation indicated that one candidate gene, *CsNCED1*, might significantly inhibit SA content. The analysis of plants treated with exogenous ABA and transgenic plants overexpressing the *CsNCED1* gene revealed that ABA could decrease the SA content and expression levels of the SA biosynthesis gene (*ICS*), SA receptor gene (*NPR1*), and NPR1 targeted genes (*PR1* and *WRKY18*) to enhance the sensitivity to biotic stress in the insect-feeding experiment. Our study elucidated the molecular regulatory mechanisms of tea plants in disease and pest defense for the first time, potentially offering a robust theoretical and practical framework for insect resistance breeding and the sustainable usage of tea plant resources.

## Materials and methods

### Tea plant material collection

This study used a collection of 299 tea accessions, which included 203 *Camellia sinensis*, 46 *Camellia tachangensis*, 46 *Camellia remotiserrata*, and 4 *Camellia taliensis* (Table S1). Of these, 295 were sourced from 30 counties in Guizhou, with the remaining four tea accessions originating from Hunan, Fujian, and Zhejiang provinces (Table S1). All plants were cultivated in a dedicated nursery at Guizhou University and uniformly managed using the tea garden’s conventional water and fertilizer management procedures.

### Statistical analysis of SA content in 299 tea accessions

For the analysis, samples of one bud and three leaves from each accession were collected in the springs of 2020 (Environment I) and 2021 (Environment II). All collected leaves were submerged in liquid nitrogen. The SA content was quantified using HPLC-1440 [44]. Statistical analysis was done using the SPSS software (v.25) from IBM Corp. (Armonk, NY, USA).

### Identification of high-quality SNPs

The extracted DNA from 299 tea samples was sequenced using Illumina’s HiSeq-X platform [45]. BWA-MEM mapped the clean data to the reference genome (http://tpia.teaplants.cn/) [46]. The Genome Analysis Toolkit (GATK, v 3.7.0) software was applied to perform the SNP calling, which were then filtered using methodologies proposed by Niu et al. and McKenna et al. [45, 47]. Plink v.1.90 was used to calculate the major allele frequency (*MAF*), expected heterozygosity (*He*), inbreeding coefficient (*Fis*), and observed heterozygosity (*Ho*) for each inferred group [48]. VCFtools (version 0.1.160) was employed to determine the nucleotide diversity and Tajima’s D for each inferred subgroup [49, 27].

### Population structure analysis

The population composition of the 299 tea accessions was determined using the ADMIXTURE program, which may be accessed at ADMIXTURE [50]. We defined a range of potential cluster numbers (K values) from 1 to 10 to determine the population structure, providing maximum likelihood estimates for each accession across all possible K populations. We used the MEGA-X software to generate a neighbor-joining (NJ) phylogenetic tree [51] and performed principal component analysis (PCA) using the Tassel software (v. 5.2). Genome-wide linkage disequilibrium (LD) values were computed by analyzing the correlation coefficient (r [2]) of paired SNPs with PopLDdecay (available at PopLDdecay) using the default parameters [52].

### GWAS analysis

GWAS was performed on 299 tea accessions using Tassel software and six different methods (GLM-P, GLM-Q, MLM-PK, MLM-QK, CMLM-PK, and CMLM-QK). To reduce potential false positives caused by population structure mixing, the GLM models used either the Q-matrix or PCA as fixed effects. In MLMs and CMLMs, the kinship matrix (K) was used as a covariate to account for unequal genotype correlations. Analyzing the Q-Q plots revealed the appropriate model for each attribute. A threshold of −Log_10_ (*P*) ≥ 4.0 [53] was utilized to detect SNPs associated with SA content.

### Prediction of candidate genes associated with SA content

Candidate genes were identified from significant high-quality SNPs selected using the best models. Potential candidate gene distribution regions were defined as genes within 15 kb upstream or downstream of linked SNP loci. The NCBI database annotated the candidate genes associated with SA content.

### Expression profiles of candidate genes and functional validation

RNA extraction and quantitative polymerase chain reaction (qPCR) analysis: One bud and two leaves from 15 tea accessions with varying SA contents (from highest to lowest) were used to extract RNA using Trizol DNA-free kits (Shanghai Shenggong Biotechnology Co. LTD.). The RNA was reverse-transcribed into cDNA, serving as the RT-qPCR template. The transcript abundances of candidate genes were detected using the 2^^(−ΔΔCt)^ method, with GAPDH as the reference gene. Each sample was replicated thrice (Table S2).

ABA spraying experiment: Two groups of tea plants with identical growth status were selected. The experimental group was sprayed with a 5 mg/ml ABA solution, whereas the control group received deionized water. The ABA solution was sprayed every 3 days, for a total of three sprays. The SA content and gene expression levels were measured using the previously described method [54].

Antisense oligonucleotide (asOND) experiment: We used Soligo software to design asOND sequences complementary to the target gene fragment, utilizing tea’s *CsNPR1* sequence as input [55]. Sense oligonucleotides (sOND) served as controls. Both sOND and asOND were dissolved in ddH_2_O at 20 OD for soaking treatments. This experiment was replicated thrice, with three buds and two leaves per repeat (Table S2).

Overexpression of *CsNCED1* in transgenic tobacco: The complete coding sequence of the *CsNCED1* gene was cloned into the Eco31I sites of the pBWA(V)HS vector. The transformed tobacco lines were selected using the leaf disc transformation method and screened with 30 mg/l hygromycin [56, 57]. PCR was used to validate a total of 11 independent transgenic lines. The WT and transgenic plants were cultured on blank MS medium and MS medium with hygromycin under controlled conditions (23 ± 2°C, 60% RH, 16 h light/8 h dark photoperiod) [58]. Following two generations, homozygous transgenic lines were selected for further analysis.

Phenotypic and biochemical analysis of transgenic lines: 8-week-old seedlings of various transgenic tobacco lines were examined for *CsNCED1* gene expression as well as ABA and SA content by HPLC-1440 [44]. Plant morphology, stem and leaf orientation, leaf size, and trichome density were also evaluated. Leaf thickness was measured using the safranin green staining method in paraffin sections [59, 60].

Insect-feeding experiment: The resistance of WT and transgenic tobacco to insect damage was evaluated using *Spodoptera litura* larvae raised under controlled conditions (26 ± 2°C, 70% ± 5% humidity, 12 h light/12 h dark). Healthy, uniform larvae were selected for the feeding trial to compare the damage levels between the plant types.

## Supplementary Material

Web_Material_uhae362

## Data Availability

The raw sequence data reported in this study are available at the Genome Sequence Archive in BIG Data Center, Beijing Institute of Genomics (BIG) (http://bigd.big.ac.cn/gsa) under accession number CRA001438.
