# Supplementary material for: Genome-wide association study of salicylic acid provides genetic insights for tea plant selective breeding
Source: Hortic Res. 2025 Jan 2;12(4):uhae362. doi: 10.1093/hr/uhae362 (PMC11891476; doi:10.1093/hr/uhae362)
Supplement: Web_Material_uhae362 [file web_material_uhae362.zip › Supplemental Figure 1-2.docx]

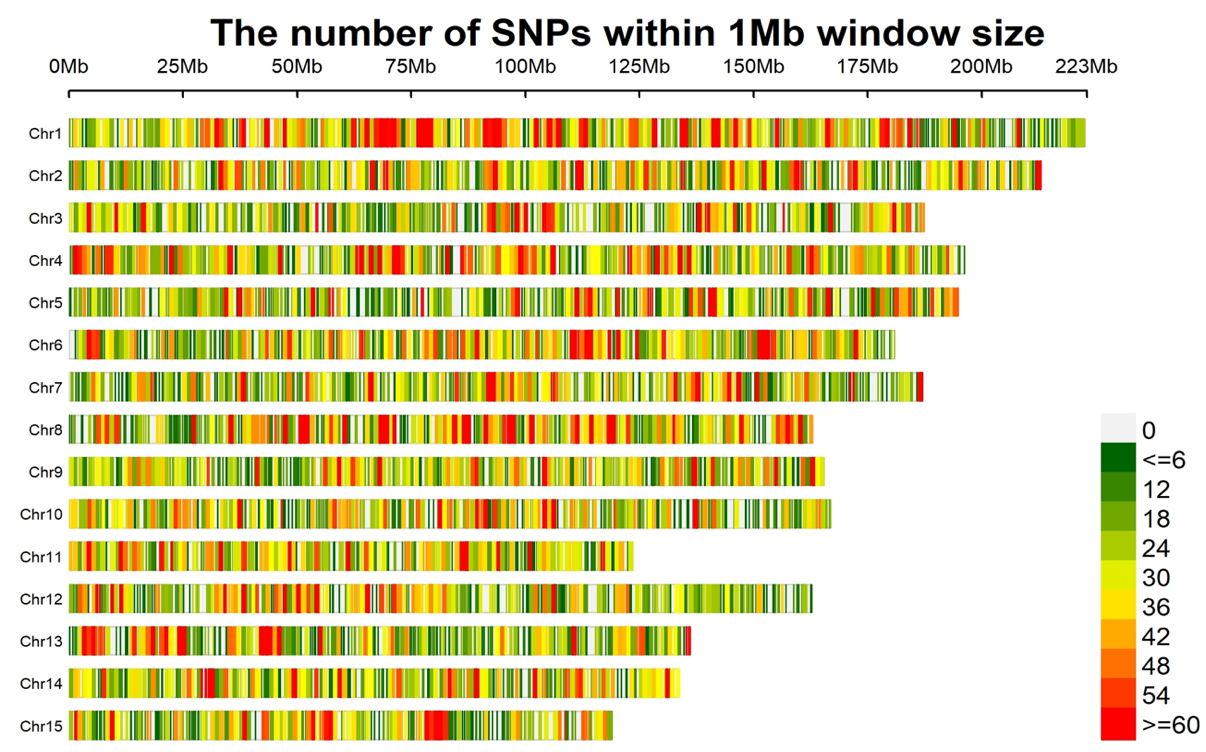


**Figure S1.** Distribution of single nucleotide polymorphisms (SNPs) on 15 chromosomes of the tea plant. The horizontal axis shows the chromosome length.


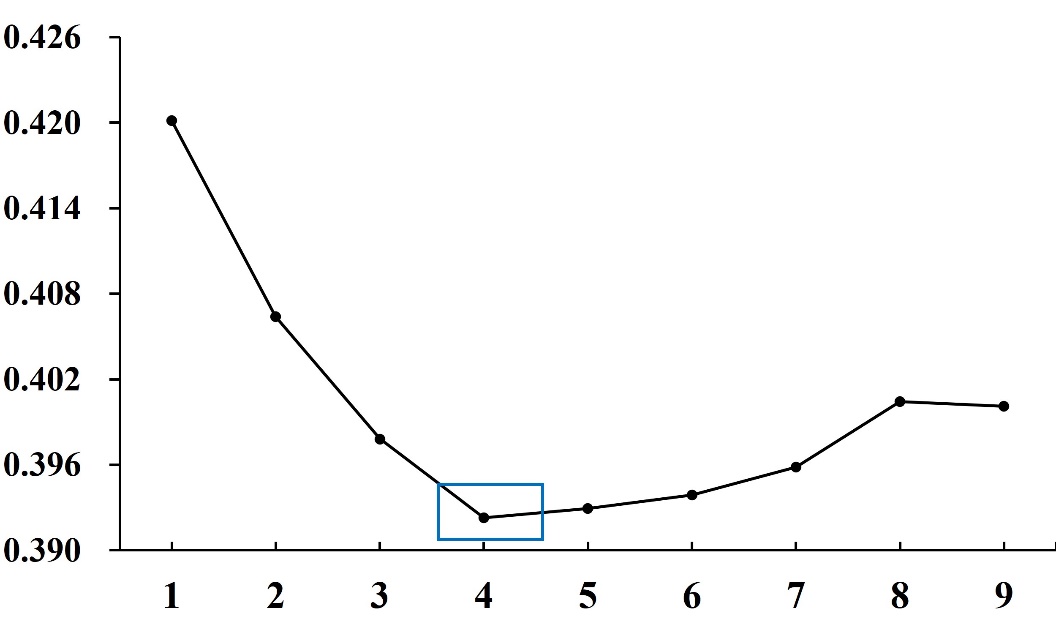


**Figure S2.** The cross-validation error (CV error) of the corresponding K = 1-9.
